# Hypertension Resistant to RAAS Inhibitors as a Prognostic Indicator for Rapid Progression to ESRD in ADPKD: A Ten-Year Follow-Up

**DOI:** 10.3390/diagnostics15202583

**Published:** 2025-10-13

**Authors:** Andrea Angioi, Doloretta Piras, Nicola Lepori, Matteo Floris, Gianfranca Cabiddu, Antonello Pani

**Affiliations:** 1 S.C.D.U. Nefrologia, Dialisi e Trapianto, ARNAS Brotzu, Piazzale Ricchi 1, 09100 Cagliari, Italy; 2 Dipartimento di Scienze Mediche e Sanità Pubblica, Università di Cagliari, 09100 Cagliari, Italy; nicola.lepori@aob.it (N.L.); matteo.floris@unica.it (M.F.); gianfranca.cabiddu@aob.it (G.C.); antonellopani@aob.it (A.P.)

**Keywords:** polycystic kidney disease, hypertension, ADPKD, chronic kidney disease, RAAS inhibitors

## Abstract

**Background:** Autosomal dominant polycystic kidney disease (ADPKD) is characterized by progressive renal cyst development and variable trajectories toward end-stage renal disease (ESRD). Hypertension is both common and prognostically significant in ADPKD. However, the escalating need for antihypertensive agents beyond RAAS inhibition on disease progression remains underexplored. **Methods:** We conducted a retrospective, single-center cohort study including 133 ADPKD patients followed for a median of 5 years. Baseline clinical, biochemical, and genetic data were collected. The primary outcome was a ≥25% decline in eGFR over 5 years. All patients achieved a blood pressure target range of 110/70 to 130/85 mmHg during follow-up. Univariate and multivariate logistic regression analyses were performed to identify predictors of rapid progression. **Results:** Patients with hypertension resistant to RAAS (i.e., those requiring additional antihypertensive drugs on top of RAAS inhibitors) had significantly higher odds of rapid eGFR decline (multivariate OR 1.27; 95% CI 1.03–1.57; *p* = 0.0248). The presence of hypertension resistant to RAAS was interpreted as a potential clinical surrogate for a more aggressive cystic phenotype and intrarenal hemodynamic dysregulation. **Conclusions:** Hypertension resistant to RAAS is an independent predictor of accelerated renal function decline in ADPKD. Its identification may aid in early risk stratification and prompt consideration of disease-modifying therapies such as tolvaptan. Further validation in larger cohorts is warranted.

## 1. Introduction

Autosomal dominant polycystic kidney disease (ADPKD) is a major health concern because it is the most common hereditary kidney disease worldwide, affecting about 12.5 million individuals [1]. Characterized by multiple cysts mainly in the kidneys and liver, ADPKD presents a significant healthcare challenge because it can progress to kidney failure, ultimately needing replacement therapy.

Approximately 85% of ADPKD cases result from mutations in the PKD1 gene, while mutations in the PKD2 gene account for about 10–15% of cases [2]. Mutations in genes involved in protein biogenesis in the endoplasmic reticulum, such as ALG9, GANAB, SEC63, SEC61B, DNAJB11, and ALG8, can also cause cystic kidney and liver diseases by disrupting the production or function of polycystin-1 [3]. These proteins are vital components of primary cilia in tubular epithelial cells, where they regulate cell growth, programmed cell death, water and electrolyte influxes and effluxes, and extracellular matrix remodeling [3]. The genetic defect initially causes the formation of cystic dilation (diffuse), and then, in less than 5% of tubules, the development of cysts that vary from microscopic to large enough to be visible on ultrasound or RMN. These cysts gradually enlarge, impairing kidney function through both mechanical pressure and functional disruption.

Despite this genetic burden, the progression of ADPKD varies greatly among individuals, with some developing end-stage renal disease (ESRD) early in life. Others, however, retain moderate kidney function into old age, with the same mutation. This variability highlights a significant gap in our understanding of ADPKD’s natural course and the epigenetics that influence disease progression [4].

In the literature, prognostic scores are used to predict, based on clinical and genetic criteria, whether an individual will have a rapid or slow progression to ESRD. The most commonly used score in clinical practice is the PRO-PKD score. This prognostic model utilizes genetic and clinical data to identify key factors, including gender, hypertension before age 35, the first urologic event before age 35, PKD2 mutation, and non-truncating or truncating PKD1 mutation. These factors enable the stratification of patients into low, intermediate, and high-risk categories, each associated with different median ages for ESRD onset [5]. Other models have been described in the literature that consider additional variables (e.g., early-onset hypertension, height-adjusted mean kidney length); notably, every score highlights the degree of genetic mutation as a critical independent predictor [6,7]. However, in real-world clinical settings, the PRO-PKD score identifies fewer rapid progressors compared to the Mayo score, and it is not universally effective across all cohorts [8].

Uncovering the factors that influence disease progression in ADPKD can provide valuable insights for customizing individual treatment strategies. Therefore, this study aimed to examine how specific clinical features and baseline biochemical parameters affect CKD progression in ADPKD patients within our single-center cohort over a 10-year follow-up period.

## 2. Materials and Methods

### 2.1. Study Design and Participants

This retrospective, a single-center cohort study was conducted at a tertiary care institution (ARNAS “G. Brotzu,” Cagliari, Italy), involving patients in a dedicated outpatient clinic specialized in Polycystic Kidney Disease and other Cystic Disorders with 172 patients on follow-up on a regular basis.

Inclusion criteria: (1) in cases of a positive family history, a diagnosis of ADPKD was based on Pei’s ultrasonographic criteria: age 15–39 years, ≥three renal cysts (unilateral or bilateral); age 40–59 years, ≥2 cysts in each kidney; age ≥ 60 years, ≥four cysts in each kidney [9]. (2) In case of a negative family history: presence of 10 or more cysts (≥5 mm) in each kidney, especially if the kidneys are enlarged or liver cysts are observed, and when there are no apparent signs of a different cystic disorder. (3) Genetic diagnosis by identifying pathogenic mutations in the PKD1 or PKD2 genes.

Exclusion criteria included (1) patients classified as atypical ADPKD (Class 2 Mayo ADPKD classification); (2) less than two documented visits; (3) insufficient medical record data for essential outcome analysis; (4) patients younger than 18 years.

Of 172 patients diagnosed and managed at our institution between January 2018 and December 2024, 133 were eligible.

### 2.2. Data Collection

Demographic, clinical, genetic, therapeutic, and biochemical data were retrospectively obtained from electronic medical records (Table 1 and Table 2). Collected baseline characteristics included age, sex, body mass index (BMI), body surface area (BSA), gene mutation (PKD1, PKD2, or unidentified mutations), extrarenal manifestations, family history, baseline kidney volume (MRI-derived), baseline kidney function parameters (serum creatinine, eGFR calculated by CKD-EPI 2021 equation, creatinine clearance), 24 h proteinuria, hypertension, antihypertensive treatments (RAAS inhibitors including ACE inhibitors or ARBs, and other antihypertensive agents), and tolvaptan. The PRO-PKD score was calculated based on genetic and clinical factors (gender, hypertension before age 35 years, urologic complications before age 35 years, PKD mutation type, and mutation characteristics). Kidney pain was defined as recurrent flank pain explicitly attributed to renal cyst enlargement or complications, documented clinically, and occurring more than twice per year.

### 2.3. Outcomes and Follow-Up

The primary outcome measured was the decline in kidney function, specifically the predicted percentage reduction in eGFR at five years, with a clinically significant threshold set at a 25% reduction [10]. The annualized eGFR slope (mL/min/1.73 m^2^ per year) was calculated for each patient to evaluate the course of kidney function decline over the follow-up period. The median follow-up duration was five years, with routine clinical and biochemical assessments conducted every 3–12 months as needed. All patients achieved guideline-concordant BP targets: ≤110/75 mmHg by HBPM in adults 18–49 with CKD G1–G2, and standardized office SBP < 120 mmHg in adults ≥50 years or with lower eGFR, as per KDIGO 2025 (ADPKD) [11]. The target was reached and monitored by a daily blood pressure control. Patients were trained by their general practitioner to follow this HBPM protocol (cuff sizing/positioning, posture and 5 min rest, morning/evening measurements, and diary recording); technique and adherence were reviewed at routine visits.

### 2.4. Statistical Analysis

Continuous variables were described as medians with interquartile ranges (IQRs) due to their non-normal distribution, as confirmed by the Shapiro–Wilk test, or as means ± standard deviations (SDs) when variables exhibited a normal distribution. Categorical variables were presented as frequencies and percentages. Differences in baseline characteristics before and after propensity score matching were evaluated using Student’s *t*-test for normally distributed variables and the Mann–Whitney U test for skewed variables, while categorical variables were compared with Chi-square or Fisher’s exact tests, as appropriate.

Univariate logistic regression analysis identified clinical and biochemical factors associated with the outocome, expressed as odds ratios (OR) with 95% CI.

All variables meeting this threshold were then simultaneously entered into a multivariate logistic regression model using the Enter (forced-entry) method in JASP (Version 0.19.3). This approach ensured that the effect of each covariate was estimated while adjusting for all others, without resorting to stepwise selection.

### 2.5. Software and Ethics

All statistical analyses were conducted using JASP software, version 0.19.3 (JASP Team, Amsterdam, The Netherlands). The retrospective study was approved by the Institutional Ethics Committee of the Azienda Ospedaliero-Universitaria di Cagliari (protocol number PG/2019/16560, approved on 27 November 2019). Informed consent was obtained through the protocol guidelines.

## 3. Results

### 3.1. Patient Demographics and Clinical Characteristics

The study included 133 patients diagnosed with ADPKD, predominantly harboring PKD1 mutations (73.68%), followed by PKD2 mutations (10.53%) and unidentified mutations (15.79%). Males comprised 44.36% of the cohort, and 85.00% reported a positive family history. Frequent extrarenal manifestations included cardiac hypertrophy (37.59%), intraductal papillary mucinous neoplasms (IPMN, 17.29%), and nephrolithiasis (8.27%). Hypertension was prevalent in 55.63%, and frequent kidney pain (>2 episodes/year) was reported by 52.63% (Table 1 and Table 2).

### 3.2. Treatment and Therapeutic Patterns

ACE inhibitors were the most commonly prescribed antihypertensive therapy, used by 64 patients (48.12%), while ARBs were given to 23 patients (17.29%). A combination of RAAS inhibitors (either ACE inhibitors or ARBs) plus additional antihypertensive medications was needed for 37 patients (27.8%). In total, 50 patients (37.6%) received only RAAS inhibitors without extra antihypertensive agents. A smaller group (7.5%, 10 patients) was managed solely with antihypertensive medications that did not include RAAS inhibitors. Tolvaptan was administered to 17 eligible patients (12.8%), whereas 36 patients (27.1%) did not receive any antihypertensive therapy due to hypotension.

### 3.3. Biochemical Parameters and eGFR Progression

The baseline median eGFR was 67.9 mL/min/1.73 m^2^ (IQR 31.0), significantly declining to 48.9 mL/min/1.73 m^2^ (IQR 32.9) at five years, reflecting a median decrease of 19.0 mL/min/1.73 m^2^ (IQR 11.89). The annual median eGFR decline was 3.8 mL/min/1.73 m^2^ per year. The baseline kidney volume, assessed by MRI, was 1379.95 mL (IQR, 881.8).

### 3.4. Identification of Predictive Factors for CKD Progression

Univariate logistic regression identified significant predictors of a 25% eGFR reduction at 5 years (Table S1). Use of RAAS inhibitors plus antihypertensive drugs was strongly associated with the progression of kidney disease (OR 3.03, 95% CI 1.16–7.914, *p* = 0.02360), as was average creatinine at first visit (OR 3.453, 95% CI 1.274–9.362, *p* = 0.01489). Initial eGFR (OR 0.969, 95% CI 0.952–0.987, *p* = 0.00059) and creatinine clearance (OR 0.971, 95% CI 0.955–0.987, *p* = 0.00037) showed protective effects. ACE inhibitors (OR 1.475, *p* = 0.39346) and ARBs (OR 1.247, *p* = 0.69460) alone were not significantly associated with progression (Table 3).

Multivariate linear regression refined these findings (Table 4). A lower initial eGFR was a significant predictor of greater eGFR decline (OR 0.993, CI 95% 0.989–0.997; *p* = 0.0012), as was the use of other antihypertensive drugs (OR 1.272, CI 95% 1.032–1.569; *p* = 0.0248). Diverticulosis and kidney pain were included in the multivariate analysis since they were close to the significance threshold in the univariate analysis but did not reach statistical significance in the multivariate model (*p* > 0.05). This approach does not account for time-to-event information, which is acknowledged as a limitation compared with Cox proportional-hazards modeling.

## 4. Discussion

**Figure 1:**
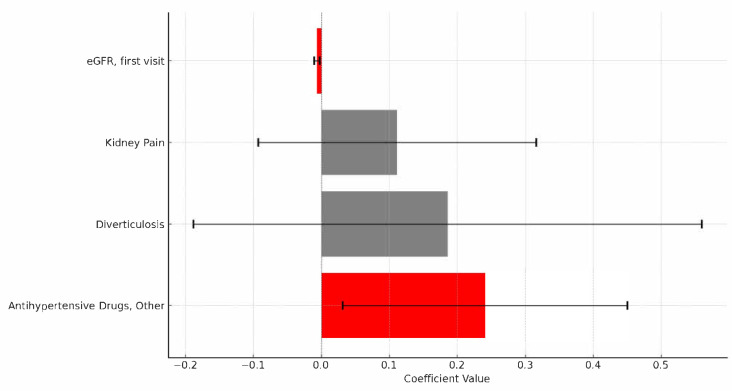
The horizontal bars represent the coefficients of each variable, which indicates the estimated effect size. The error bars show the 95% confidence intervals for each coefficient. Variables highlighted in red have *p*-values below 0.05, suggesting they are statistically significant at the 5% level. From the plot: The variable “eGFR, first visit” has a negative and statistically significant coefficient, suggesting a negative relationship with the dependent variable. The variable “Antihypertensive Drugs, Other” has a positive coefficient and is statistically significant, suggesting a positive relationship with the dependent variable. The other variables are not statistically significant at the 0.05 level.

In this single-center cohort of ADPKD patients, we found that hypertension resistant to RAAS, operationally defined as the need for additional antihypertensive therapy beyond RAAS inhibition, is a significant marker of rapid disease progression. Patients who required more than a single RAAS blocker to control blood pressure had higher odds of experiencing a ≥25% decline in eGFR over 5 years. On multivariate analysis adjusting for key baseline variables (including age, sex, and baseline eGFR), hypertension resistant to RAAS remained an independent predictor of kidney function decline (adjusted OR 1.27, 95% CI 1.03–1.57; *p* = 0.0248) (Figure 1). These findings suggest that difficulty in achieving blood pressure control, despite optimal first-line therapy, is not merely a coincidental comorbidity but rather a proxy for a more aggressive cystic disease. This is biologically plausible: extensive cyst burden in ADPKD activates the intrarenal RAAS, leading to early and severe hypertension. Indeed, the severity of hypertension correlates with TKV and cyst growth rate in ADPKD. In clinical terms, hypertension resistant to RAAS serves as a red flag for clinicians, identifying patients with ADPKD who are on a trajectory of rapid renal function loss [12,13].

Our results align with and extend prior observations regarding hypertension and ADPKD progression. It has long been recognized that hypertension is highly prevalent in ADPKD and portends worse outcomes [14]. Approximately 50–70% of ADPKD patients develop hypertension before any significant loss of GFR, often decades earlier than in hypertensive patients without ADPKD. Early-onset hypertension in ADPKD is strongly associated with faster cyst growth and earlier onset of end-stage renal disease (ESRD). Previous longitudinal studies have identified baseline hypertension as an independent risk factor for kidney function decline and progression to ESRD in ADPKD [15,16]. Notably, the PROPKD score incorporates “early hypertension” (onset before age 35) as a key risk factor, along with gene mutation type and urologic complications [5].

Our findings build upon this knowledge by focusing on the subset of patients with hypertension to RAAS. While prior studies emphasized the presence or absence of hypertension, we show that the degree of blood pressure control (or lack thereof) on standard therapy carries additional prognostic significance. In other words, it is not only whether an ADPKD patient is hypertensive, but how difficult that hypertension is to treat, that predicts renal outcome. This nuance has not been explicitly addressed in earlier ADPKD cohorts and provides a new perspective on risk stratification.

The results of our study are also consistent with broader observations in chronic kidney disease (CKD) populations. In non-ADPKD CKD, resistant or poorly controlled hypertension has been linked to accelerated renal function loss and higher rates of reaching ESRD [15].

The clinical implications of these findings are immediate and practical. First and foremost, hypertension resistant to RAAS, if confirmed in other cohorts, should be recognized as a high-risk feature in ADPKD. In routine practice, it is relatively easy to identify patients who meet this criterion (those already on an ACE inhibitor or ARB who require one or more additional drugs) to achieve blood pressure targets. Such patients should be flagged for enhanced risk stratification and closer follow-up, prompting clinicians to actively evaluate for rapid disease progression. This could include obtaining baseline and follow-up measurements of TKV (e.g., via MRI) sooner, to see if the patient falls into a high-risk Mayo class. It could also include considering genetic testing if not already done, since a patient with difficult-to-control hypertension may have an underlying high-impact PKD1 mutation or other genetic modifiers of severity. By incorporating hypertension resistant to RAAS into the risk assessment, clinicians can identify “rapid progressors” earlier in the disease course. This aligns closely with KDIGO 2025 guidance, which stresses early identification of patients likely to benefit from therapy to delay progression [11]. Another significant implication is the potential to initiate disease-modifying treatments earlier in patients with hypertension resistant to RAAS. The vasopressin V2-receptor antagonist tolvaptan is currently the only approved therapy shown to slow ADPKD progression. Tolvaptan has been demonstrated in large trials to slow the rate of increase in TKV and the decline in GFR in ADPKD [16,17].

However, we acknowledge several significant limitations of our cohort. (1) The study’s retrospective observational design inherently limits causal inferences. (2) The lack of comprehensive genetic data (15.79% of patients have an unknown mutation). (3) It was conducted at a single tertiary care center, limiting generalizability. (4) Our criterion of “beyond RAAS inhibition” captures a slightly broader group, potentially including some patients who needed just a second agent. (5) Non-adherence may have played a role. (6) As with any observational study, there may be residual confounders. (7) The outcome we used, a 25% decline in eGFR, while clinically meaningful, is still a surrogate endpoint of “hard” endpoints like ESRD, but our cohort’s follow-up (median 5.2 years) and sample size yield relatively few of those events to power the analysis.

## 5. Conclusions

In this single-center cohort of ADPKD patients, hypertension resistant to RAAS, defined as the need for additional agents beyond RAAS inhibition, emerged as an independent predictor of accelerated eGFR decline over 5 years. Its presence may reflect a more aggressive cystic burden and intrarenal hemodynamic dysregulation. Early identification of hypertension resistant to RAAS could prompt intensified monitoring and earlier initiation of disease-modifying interventions. Validation in larger cohorts is warranted.

## Figures and Tables

**Table 1:** Baseline Characteristics of the Study Population—Categorical variables (*N* = 133).

Variable	No. (%)
Male Sex	59 (44.36)
Family History	113 (85.00)
PKD Gene	
PKD1	98 (73.68)
PKD2	14 (10.53)
No Mutation Identified	21 (15.79)
Extrarenal Manifestations	
Cerebral Aneurysm	2 (1.50)
Pulmonary Disease	3 (2.26)
Nephrolithiasis (Kidney Stones)	11 (8.27)
IPMN *	23 (17.29)
Cardiac Hypertrophy	50 (37.59)
Cardiac Valve Defect	8 (6.02)
Diabetes Mellitus	3 (2.26)
Diverticulosis	5 (3.76)
Kidney Pain (>2 episodes/year)	70 (52.63)
Hernia	9 (6.77)
**Risk Factors for End-Stage Kidney Disease**	
Smoking	14 (10.53)
Cyst Infection (per year)	19 (14.29)
Hypertension	74 (55.63)
Urinary Tract Infection	39 (29.32)
Hematuria	38 (28.57)
Nephrectomy	2 (1.50)
Mayo Classification for ADPKD	
Mayo 1A	33 (24.81)
Mayo 1B	46 (34.59)
Mayo 1C	27 (20.30)
Mayo 1D	16 (12.03)
Mayo 1E	11 (8.27)
Treatments	
ACE Inhibitors	64 (48.12)
ARBs	23 (17.29)
Other Antihypertensive Drugs	10 (7.5)
No Antihypertensive	36 (27.1)
RAAS + Other Antihypertensives	37 (27.8)
Tolvaptan	17 (12.80)
Proteinuria > 300 mg/24 h, Baseline	33 (24.81)
**Proteinuria > 300 mg/24 h, Last Visit**	49 (36.84)

Abbreviations: PKD, Polycystic Kidney Disease; ACE, Angiotensin-Converting Enzyme; ARB, Angiotensin II Receptor Blocker; eGFR, estimated Glomerular Filtration Rate; ADPKD, Autosomal Dominant Polycystic Kidney Disease; RAAS, Renin–Angiotensin–Aldosterone System. * IPMN = Intraductal Papillary Mucinous Neoplasm.

**Table 2:** Baseline Characteristics of the Study Population—Continuous variables (*N* = 133).

Variable	Median (IQR)
Age (years)	52.1 (13.2)
Height (cm)	165.89 (7.95)
Weight (kg)	65.2 (10.9)
**Body Mass Index (kg/m^2^)**	23.74 (7.41)
Body Surface Area (m^2^)	1.7268 (0.1497)
**Kidney Volume on MRI (mL)**	1379.95 (881.8)
**Annual eGFR slope (mL/min/1.73 m^2^)**	3.8 (2.38)
**eGFR (first visit) (mL/min/1.73 m^2^)**	67.9 (31.0)
**eGFR (last visit) (mL/min/1.73 m^2^)**	48.9 (32.9)
**eGFR slope (mL/min/1.73 m^2^) in 5 years**	19.0 (11.89)
**PRO-PKD score, median (IQR)**	4 (2–6)

**Table 3:** Univariate analysis of covariates (outcome: 25% predicted GFR reduction after five years of follow-up).

Covariate	OR	Lower CI (95%)	Upper CI (95%)	*p*-Value
ACEI	1.475	0.604	3.603	0.39346
Age	1.029	0.994	1.065	0.10244
**Antihypertensive Drugs, Other** ^†^	**3.03**	**1.16**	**7.914**	**0.02360**
ARBS	1.247	0.415	3.749	0.69460
Body Mass Index	1.026	0.933	1.129	0.59939
Body Surface Area	1.099	0.059	20.584	0.94980
Cyst Infection	1.458	0.379	5.614	0.58329
Diabetes	2.923	0.291	29.356	0.36211
Diverticulosis	6.333	0.726	55.221	0.09480
Estimated Heart Risk	1.029	0.994	1.065	0.10365
**eGFR, first visit**	**0.969**	**0.952**	**0.987**	**0.00059**
Hernia	4.0	0.427	37.459	0.22451
Hypertension	1.96	0.767	5.01	0.15963
htTKV	1.0	0.999	1.001	0.66333
Hypertension on drugs	1.552	0.622	3.87	0.34593
Kidney Pain	2.336	0.958	5.697	0.06218
Kidney Volume to Body Surface Area	1.0	0.999	1.001	0.65432
MRI kidney volume	1.0	0.999	1.0	0.67345
Proteinuria, Time 0	0.682	0.187	2.48	0.56100
Macrohematuria, recurrent	0.655	0.227	1.889	0.43313
Smoking	0.893	0.357	2.232	0.80847
Tolvaptan	1.1	0.335	3.614	0.87522
Urinary Tract Infection	1.603	0.574	4.482	0.36799
Weight	1.005	0.966	1.046	0.79594

Abbreviations: OR, odds ratio; CI, confidence interval. ^†^ Antihypertensive agents added on top of RAAS blockade (ACE Inhibitors or ARBs).

**Table 4:** Multivariate analysis of covariates (outcome: 25% predicted GFR reduction at 5 years).

Variable	OR	95% CI (Upper)	95% CI (Lower)	*p*-Value
eGFR, first visit	0.993	0.989	0.997	**0.0012**
Antihypertensive Drugs, Other ^†^	1.272	1.032	1.569	**0.0248**
Diverticulosis	1.204	829	1.75	0.3255
Kidney Pain	1.118	911	1.372	0.2810

Abbreviations: eGFR, estimated glomerular filtration rate. ^†^ Additional blood-pressure–lowering agents added on top of RAAS blockade (ACE Inhibitors or ARBs).

## Data Availability

The individual-level clinical (and, where applicable, genetic) data underlying this article contain potentially identifiable health information and are therefore not publicly available in order to comply with GDPR and institutional privacy policies. De-identified data and a data dictionary can be provided on reasonable request to the corresponding author.

## Supplementary Materials

The following supporting information can be downloaded at: https://www.mdpi.com/article/10.3390/diagnostics15202583/s1.
