# Supplementary material for: Hypertension Resistant to RAAS Inhibitors as a Prognostic Indicator for Rapid Progression to ESRD in ADPKD: A Ten-Year Follow-Up
Source: Diagnostics (Basel). 2025 Oct 13;15(20):2583. doi: 10.3390/diagnostics15202583 (PMC12564625; doi:10.3390/diagnostics15202583)
Supplement: Supplementary file 1 [file diagnostics-15-02583-s001.zip › diagnostics-3841251-supplementary.pdf]

Table S1a. Baseline characteristics according to outcome (≥25% eGFR decline at 5 years vs no decline) – Continuous variables.

Variable	With outcome (n = 60)	Without outcome (n = 73)
Age, years	54 [48–60]	50 [43–57]
Height, cm	166.2 [161.0–171.5]	165.6 [160.5–170.8]
Weight, kg	67.8 [60.5–75.4]	63.4 [57.9–70.1]
Body Mass Index, kg/m²	24.6 [22.1–27.8]	23.1 [21.0–25.2]
Body Surface Area, m²	1.76 [1.67–1.86]	1.70 [1.62–1.79]
Kidney Volume on MRI, mL	1,680 [1,240–2,160]	1,120 [880–1,460]
**eGFR (first visit), mL/min/1.73 m² ‡**	**52** [42–64]	**81** [67–91]
eGFR (last visit), mL/min/1.73 m²	32 [24–44]	64 [55–74]
Annual eGFR decline, mL/min/1.73 m²/yr	5.1 [4.2–6.1]	2.8 [2.1–3.4]
Absolute eGFR decline in 5 yrs, mL/min/1.73 m²	26 [22–30]	12 [9–15]
PRO-PKD score	5 [3–7]	3 [2–5]

Table S1b. Baseline characteristics according to outcome (≥25% eGFR decline at 5 years vs no decline) – Categorical variables.

Variable	With outcome (n = 60)	Without outcome (n = 73)
**Male sex**	29 (48.3%)	30 (41.1%)
**Family history**	50 (83.3%)	63 (86.3%)
**PKD gene**		
PKD1	47 (78.3%)	51 (69.9%)
PKD2	5 (8.3%)	9 (12.3%)
No mutation identified	8 (13.3%)	13 (17.8%)
**Extrarenal manifestations**		
Cerebral aneurysm	1 (1.7%)	1 (1.4%)
Pulmonary disease	1 (1.7%)	2 (2.7%)
Nephrolithiasis	6 (10.0%)	5 (6.8%)
IPMN	13 (21.7%)	10 (13.7%)
Cardiac hypertrophy	28 (46.7%)	22 (30.1%)
Cardiac valve defect	5 (8.3%)	3 (4.1%)
Diabetes mellitus	2 (3.3%)	1 (1.4%)
Diverticulosis	4 (6.7%)	1 (1.4%)
Kidney pain (>2 episodes/yr)	38 (63.3%)	32 (43.8%)
Hernia	6 (10.0%)	3 (4.1%)
**Risk factors for ESKD**		
Smoking	6 (10.0%)	8 (11.0%)
Cyst infection (per year)	10 (16.7%)	9 (12.3%)
Hypertension	38 (63.3%)	36 (49.3%)
Urinary tract infection	20 (33.3%)	19 (26.0%)
Hematuria (macro, recurrent)	17 (28.3%)	21 (28.8%)
Nephrectomy	1 (1.7%)	1 (1.4%)
**Mayo ADPKD class**		
1A	10 (16.7%)	23 (31.5%)
1B	18 (30.0%)	28 (38.4%)
1C	14 (23.3%)	13 (17.8%)
1D	10 (16.7%)	6 (8.2%)
1E	8 (13.3%)	3 (4.1%)
**Treatments**		
ACE inhibitors	34 (56.7%)	30 (41.1%)

ARBs	12 (20.0%)	11 (15.1%)
Other antihypertensive drugs (non-RAAS)	7 (11.7%)	3 (4.1%)
No antihypertensive	10 (16.7%)	26 (35.6%)
**RAAS + other antihypertensives‡**	**30 (50.0%)**	**7 (9.6%)**
Tolvaptan	9 (15.0%)	8 (11.0%)
**Proteinuria >300 mg/24h**		
Baseline	19 (31.7%)	14 (19.2%)
Last visit	35 (58.3%)	14 (19.2%)

Abbreviations: IPMN, intraductal papillary mucinous neoplasm; RAAS, renin–angiotensin–aldosterone system; ARB, angiotensin-receptor blocker; ACE, angiotensin-converting enzyme; ESKD, end-stage kidney disease.

‡ Significant at univariate analysis
